# The First Step in Adoptive Cell Immunotherapeutics: Assuring Cell Delivery via Glycoengineering

**DOI:** 10.3389/fimmu.2018.03084

**Published:** 2019-01-11

**Authors:** Robert Sackstein

**Affiliations:** Department of Translational Medicine, Herbert Wertheim College of Medicine, Florida International University, Miami, FL, United States

**Keywords:** E-selectin ligand, adoptive cell therapy, CAR T cell, GPS, sialyl Lewis X, sLeX, fucosyltransferase, translational glycobiology

## Abstract

Despite decades of intensive attention directed to creation of genetically altered cells (e.g., as in development of chimeric antigen receptor (CAR) T-cells) and/or to achieve requisite *in vitro* accumulation of desired immunologic effectors (e.g., elaboration of virus-specific T cells, expansion of NK cells, differentiation of dendritic cells, isolation, and propagation of Tregs, etc.), there has been essentially no interest in the most fundamental of all hurdles: assuring tissue-specific delivery of administered therapeutic cells to sites where they are needed. With regards to use of CAR T-cells, the absence of information on the efficacy of cell delivery is striking, especially in light of the clear association between administered cell dose and adverse events, and the obvious fact that pertinent cell acquisition/expansion costs would be dramatically curtailed with more efficient delivery of the administered cell bolus. Herein, based on information garnered from studies of human leukocytes and adult stem cells, the logic underlying the use of cell surface glycoengineering to enforce E-selectin ligand expression will be conveyed in the context of how this approach offers strategies to enhance delivery of CAR T-cells to marrow and to tumor beds. This application of glycoscience principles and techniques with intention to optimize cell therapeutics is a prime example of the emerging field of “translational glycobiology.”

## Introduction

Imagine that a product manufacturer (or vendor of the item) must make multiple shipments of the same item to a given recipient because the delivery system is neither accurate nor efficient, i.e., the physical transfer of that product to the intended arrival destination is imprecise. Such transit-related loss of goods would require that far more product be manufactured than would be needed. The faulty transport would thus be a key driver of excessive production expenses, let alone recipient costs.

Cancer treatment has entered an era whereby tumor-specific immunocytes can be created and expanded *ex vivo*, and can thereafter be administered to patients. The development of chimeric antigen receptor (CAR) T-cells is a salient example of this approach, and these antigen-specific cells have the immense advantage of achieving MHC-independent cytotoxicity of tumor targets. Once cell numbers sufficient for treatment are generated, the cells are infused into patients and serve as living drugs. To date, this approach has shown great promise in the treatment of hematologic malignancies (particularly, malignancies of B-cell origin) and is gaining applicability in solid malignancies. Yet, remarkably, in the development of such cell-based immunotherapeutics, an essential prerequisite has been uniformly overlooked: tumor regression is critically dependent on the ability of infused effector cells to enter the tumor parenchyma (1–4).

Fundamentally, it is important to draw a distinction between tissue-specific recruitment of administered cells (homing) vs. retention of administered cells at a target site. The former reflects explicit migration of cells to the intended site, whereas the latter reflects the entrapment of cells. In the case of CAR-T cells, entrapment occurs when cells that have entered a site non-specifically become retained/lodged within that tissue upon encountering their cognate antigen. Operationally, killing of malignant cells by tissue-resident T cells would ensue regardless of whether administered cells have homed to lesional sites or have entrapped there. However, entrapment is a stochastic process, and treatment efficacy could be much improved if cells were capable of homing to the affected site. For the case of CD19-directed CAR-T cells, especially for their application in acute lymphoblastic leukemia, it would be desired for administered cells to preferentially home to bone marrow. However, to date, no preclinical nor clinical studies have evaluated the extent to which administered CD19-directed CAR T-cells migrate to marrow. Instead, all past and current applications of CAR T-cells have focused on administrating sufficient quantities of cells in order to achieve the anticipated cancer treatment effect(s), with no attention to the overt waste of such cells within unaffected sites and/or the biologic consequence(s) related to off-target distribution. The inefficiency of intra-tumoral cell delivery, apart from simply requiring an exceedingly abundant cell expansion *ex vivo*, results in accumulations of cells in non-lesional sites/unaffected tissues resulting in significant treatment-related toxicities. As such, particularly for the case of CAR T-cell therapeutics, the impact of “loss of goods” should not be considered simply in terms of production expenditures, it must be factored with highest attention to the incidence of toxicities and significant patient suffering that further compound treatment-related costs. Ideally, the infused cells should not result in serious complications or, worse, mortality, but life-threatening toxicities are routine with current CAR T-cell therapy and their severities correlate with the infused cell dose (5–10).

## Circulating Lymphocyte Counts, The Car T-Cell Dose Range, and Adverse Events Associated With Car T-Cell Administration

In humans, total blood volume averages 8% of total body weight (e.g., a 50 kg person has ~4 L of blood volume). The usual lymphocyte count in humans under steady-state (healthy) conditions ranges from 1 × 10^9^ to 3 × 10^9^ cells/L. In clinical trials to date, the infusion dose of CAR T-cells has typically ranged from upwards of 2 × 10^6^-2 × 10^7^ cells/kg of recipient body weight (e.g., reflecting a dose range of 10^8^ cells to 10^9^ cells for a 50 kg person). Because this cell bolus is distributed within the total blood volume, the intravascular T-cell count immediately post-infusion ranges from 25 × 10^6^/L to 250 × 10^6^/L (please note that the conversion factor for cell dose in cells/kg into cells/L of blood volume is 12.5). Importantly, all patients that receive CAR T-cells are given lymphodepleting chemotherapy prior to the cell infusion. In essence, then, the overwhelming majority of circulating lymphocytes post-infusion are CAR T-cells, and the resulting cell count reflects as much as one-fourth the number of lymphocytes that would natively be present in the blood of a healthy person (i.e., 0.25 × 10^9^ lymphocytes/L, where normal count is 10^9^ lymphocytes/L). There is no precedent in any physiologic immune response for a circulating lymphocyte pool that is comprised predominantly (if not solely) of cells with mono-specificity for a given antigen, especially encompassing lymphocytes bearing receptors and costimulatory motifs that uniformly trigger cell activation upon encountering the cognate antigen.

The most frequent clinical adverse event associated with CAR T-cell infusions is a condition known as “cytokine release syndrome” (CRS), which is consequent to T cell activation. CRS encompasses a spectrum of clinical features including fevers, third-spacing of fluid, hypotension, and hypoxia. This constellation of physical changes is incited by release of inflammatory cytokines such as IL-6 and γ-interferon, and it can be managed by agents that block IL-6 (e.g., tocilizumab, an antibody directed to the interleukin-6 receptor), and, if necessary, steroids (6, 11). Though infrequent, CRS can progress to frank respiratory failure and other severe organ toxicities (e.g., cardiac failure, hepatitis, renal failure), requiring intensive care support (e.g., intubation/ventilatory care, vasopressors, hemodialysis), sometimes culminating in death due to organ failure. In addition to CRS, neurotoxicity known as “CAR-related encephalopathy syndrome” (CRES) can ensue, characterized by mental status changes (somnolence and/or agitation with confusion/disorientation), which can progress to increased intracranial pressures, seizures, motor weakness, and coma. As in the case of severe CRS, steroids are utilized in therapy for management of life-threatening CRES but blockade of IL-6 is ineffective in treatment of CRES, perhaps because this entity is driven by CNS infiltration of CAR T-cells (11, 12). In this regard, the potency of steroids may reflect the ability of these agents to interrupt lymphocyte trafficking (13). Importantly, though steroids yield beneficial anti-inflammatory effects, these agents can also dampen the effectiveness of the CAR T-cell assault on tumor cells.

The severity of CRS and CRES correlates principally with the dose of CAR T-cells administered, but is also related to the tempo of the *in vivo* expansion of the CAR T-cells and the extent of CAR T-cell expansion, processes that each reflect both the initial cell dose and the tumor burden of the recipient. In any case, since the localization of CAR T-cells in off-target tissues contributes to the observed organ toxicities (5, 11, 12), it is reasonable to speculate that improving the specificity of CAR T-cell infiltration within tumor sites would lessen the onset and severity of both CRS and CRES. There is strong evidence in support of this notion, as the presence of CAR T-cells in cerebrospinal fluid is correlated with the severity of CRES (12). Moreover, in preclinical studies (14–16) and in a clinical trial (17), administration of CAR T-cells directly into cancer sites has yielded marked anti-tumor effects. Importantly, in preclinical studies, the efficacy of CAR T-cells directly injected into tumor sites is much greater than that of intravenous injection (14–16), with as much as 10-fold greater cells needed intravenously to obtain equivalent anti-tumor effects (16). In the clinical trial of CAR T-cell regional administration, high doses (10^7^ cells) were administered locally without manifestations of severe systemic toxicities (17). Thus, to optimize the therapeutic window of intravascularly systemically administered CAR T-cells, it is first necessary to develop strategies to program a more precise delivery of systemically administered CAR T-cells to the relevant tumor site(s).

## The Molecular Basis of Cell Trafficking

Host defense critically depends on the capacity to ensure rapid and precise delivery of leukocytes to inflammatory sites. To this end, circulating leukocytes express a highly specific set of molecular effectors that engage endothelial cells within sites of tissue injury/inflammation. The first hurdle in all transmigration events involves the initial tethering and then rolling attachment of circulating cells to target endothelium with sufficient strength to overcome the prevailing forces of hemodynamic shear (18). This “Step 1” braking interaction is principally mediated by selectins (E-, P-, and L-selectin; known as CD62E, CD62P, and CD62L, respectively) and their ligands. Following this initial endothelial engagement, a cascade of events occur whereby cells undergo chemokine-mediated activation of integrin adhesiveness (Step 2), followed by integrin-mediated firm adherence to the endothelium (Step 3), finally resulting in transmigration (Step 4) (18).

**Figure 1:**
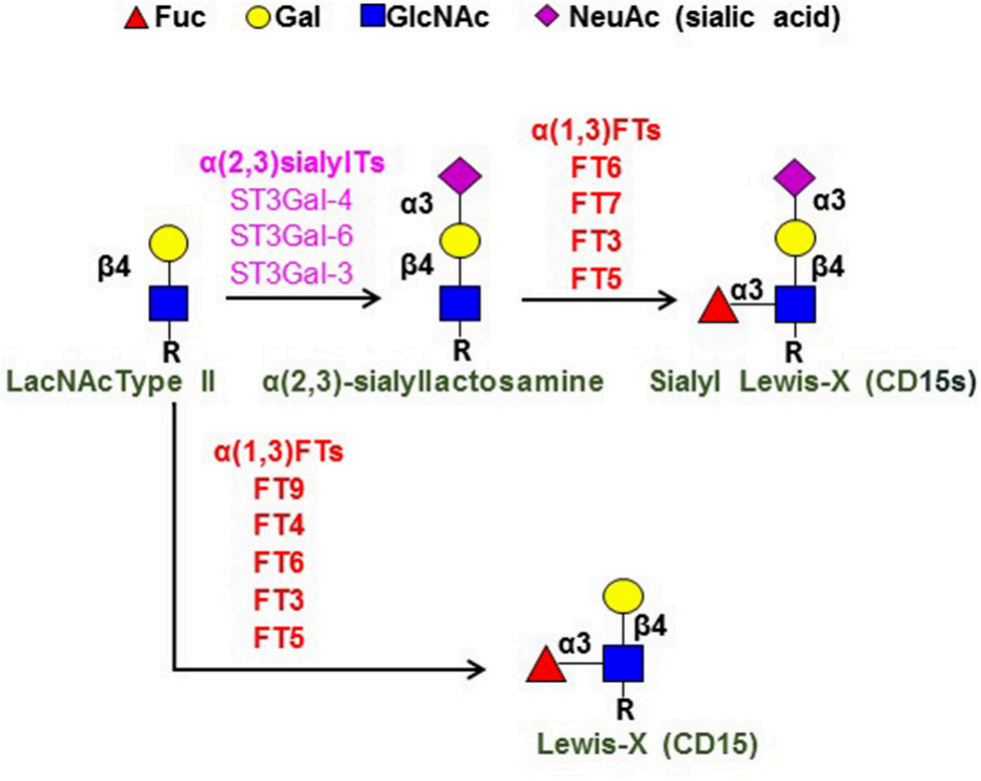
Terminal lactosamine structures. Depicted are the structures for terminal sialylated Type 2 lactosamine (LacNAc), sialylated Lewis X (sLe^X^; CD15s), and Lewis X (Le^X^; C15). Component monosaccharides are shown using colored symbol nomenclature (key is at top of figure). Shown at left is the Type 2 lactosamine unit (LacNAc Type II), a disaccharide comprised of Gal β(1,4)-linked to GlcNAc. “R” refers to the reducing end glycans, which are typically comprised of polylactosamine chains (i.e., repeating units of Type 2 lactosamines). The key enzymes in creation of sLe^X^ [the α(2,3)-sialyltransferases (α(2,3)sialylTs) and α(1,3)-fucosyltransferases (α(1,3)FTs) are as shown, as are the α(1,3)FTs that create Le^X^; these enzymes are ordered (top to bottom, high-low) to depict the relative activity of each enzyme in creating the pertinent structure [see reference (19) for details].

As indicated by their nomenclature, the selectins are “lectins,” i.e., proteins that bind to carbohydrates. This family of lectins require Ca^++^ to bind their target (i.e., the selectins are Ca^++^-dependent lectins). The prototypical carbohydrate binding determinant for all selectins is a terminal sialofucosylated lactosaminyl glycan known as “sialyl Lewis X” (CD15s) (Figure 1). This tetrasaccharide consists of a “core” disaccharide composed of the monosaccharides galactose (Gal) and N-acetylglucosamine (GlcNAc), which are joined in β(1,4)-linkage [this disaccharide is called a “Type 2” lactosamine unit (LacNAc)] (see Figure 1). The sLe^X^ determinant contains sialic acid [also known as “neuraminic acid (Neu5Ac)] that is α(2,3)-linked to the Gal, and fucose (Fuc) that is α(1,3)-linked to the GlcNAc: Neu5Ac-α(2,3)-Gal-β(1,4)-[Fuc-α(1,3)-]GlcNAcβ1-R (18). This glycan is created by step-wise addition of sialic acid and then fucose onto the terminal type 2 lactosamine core structure by respective glycosyltransferases (see Figure 1), and it is recognized by a variety of monoclonal antibodies (mAbs), including the mAb known as “CSLEX-1” and another mAb known as “HECA452.” Compared to HECA452, the CSLEX-1 mAb has a more restricted specificity in that it recognizes only sLe^X^, whereas HECA452 recognizes both sLe^X^ and the isomeric sialofucosylated type 1 lactosaminyl glycan known as sialylated Lewis A (sLe^A^). These mAb do not react with the unsialylated glycans known as “Lewis X” (Le^X^) and “Lewis A” (Le^A^) even though they share a common trisaccharide core structure with sLe^X^ and sLe^A^, respectively. Notably, the Le^X^ determinant is best known by its CD designation (“CD15”), and it is a key marker of human myeloid cells (see Figure 1).

E- and P-selectin are expressed on vascular endothelium (P-selectin also on platelets), and L-selectin is expressed on circulating leukocytes (18). E- and P-selectin are typically inducible endothelial membrane molecules that are prominently expressed at sites of tissue injury and inflammation. However, the microvasculature of bone marrow and skin constitutively expresses these selectins, and they play a key role in steady-state recruitment of blood-borne cells to these sites (20). Importantly, within all inflammatory sites and sites of tissue injury/damage in primates (but not rodents), E-selectin is the principal vascular selectin mediating cell recruitment, as the promoter element responsive to the inflammatory cytokines TNF and IL-1 has been deleted from the primate P-selectin gene. Thus, at all inflammatory sites of humans (including tumor endothelial beds), vascular E-selectin expression is more pronounced than that of P-selectin, and E-selectin also has higher baseline expression than P-selectin in human marrow and skin (18, 20).

Whereas, both glycolipids and glycoproteins can be decorated with sLe^X^ determinants, glycoproteins serve as the primary E-selectin ligands under blood flow conditions since they extend farther from the surface of the circulating cell than do glycolipids. There are three principal ligands for E-selectin expressed on subsets of human lymphocytes, each consisting of highly sialofucosylated glycoforms of well-recognized glycoproteins: CD162 (PSGL-1), CD43, and CD44. CD44 is a rather ubiquitous cell membrane protein and is best known for binding hyaluronic acid. However, display of sLe^X^ on CD44 confers new biology, and this specialized CD44 glycovariant, first observed on human hematopoietic stem/progenitor cells (HSPCs), is known as “Hematopoietic Cell E-/L-selectin Ligand” (HCELL) (21–23). As the name indicates, HCELL binds both E-selectin and L-selectin, and *in vitro* assays of E- and L-selectin binding under hemodynamic shear stress indicate that HCELL is the most potent ligand for these molecules expressed on any human cell. Notably, studies using human mesenchymal stem cells have shown that HCELL functions as a bone marrow “homing receptor” (24). Moreover, HCELL is not natively expressed on murine cells, and thus HCELL plays a uniquely prominent role in mediating human, but not mouse, HSPC migration into marrow (25).

E-selectin ligands are natively expressed on a restricted subset of human CD4 and CD8 lymphocytes, and are conspicuously absent on human B cells. However, α(2,3)-sialylated type 2 lactosamines [Neu5Ac-α(2,3)-Gal-β(1,4)-GlcNAcβ1-R] (Figure 1) are characteristically displayed on both human CD4 and CD8 cells, and, therefore, assembly of sLe^X^ on human lymphocytes pivots on α(1,3)-fucosylation of the sialylated LacNAc “acceptor” structure, i.e., the only component missing is α(1,3)-linked fucose modification of N-acetylglucosamine (GlcNAc). Importantly, sLe^X^ can only be created by fucosylation of sialylated LacNAc, as there is no mammalian sialyltransferase that can place sialic acid in α(2,3)-linkage to Gal in Le^X^ to create sLe^X^. Thus, the terminal, rate-limiting biosynthetic step for assembly of Le^X^ and sLe^X^ in each case involves fucose addition to either an unsialylated LacNAc (for Le^X^ biosynthesis) or to sialylated LacNAc (for sLe^X^ biosynthesis) (see Figure 1). This “terminal” reaction is programmed by glycosyltransferases known as α(1,3)-fucosyltransferases [α(1,3)-FTs]. In humans, there are six α(1,3)-FT isoenzymes (known as FT3, FT4, FT5, FT6, FT7, and FT9), and four of these are specialized to create sLe^X^: FT3, FT5, FT6, and FT7 (19). Of these enzymes, FT7 is the one that characteristically drives expression of sLe^X^ on human leukocytes, including lymphocytes (18, 26).

## Glycoengineering the Expression of E-Selectin Ligands: Implications for Adoptive Immunotherapeutics

Human T cells typically display high cell surface expression of CD44, CD43, and PSGL-1, the glycoproteins that can serve as scaffolds for decoration with sLe^X^ (i.e., that function as E-selectin ligands) (27). However, compared to monocytes and neutrophils that uniformly express E-selectin ligands, only a limited fraction of circulating T cells display E-selectin binding activity (27), and their E-selectin binding characteristically drops during culture-expansion in serum-containing medium (26, 28). Importantly, the absence of sLe^X^ expression on lymphocyte CD44, CD43 and PSGL-1 is solely a function of underfucosylation, as these proteins display copious amounts of terminal sialylated Type 2 LacNAc motifs (27). Indeed, the levels of sialylated LacNAc typically increase during culture-expansion of human T cells and dendritic cells (28, 29). Accordingly, installation of Fuc in α(1,3)-linkage onto GlcNAc completes the creation of sLe^X^ on the surface of the cultured cells. This cell surface glycoengineering can be achieved by introduction of nucleic acid encoding the relevant α(1,3)-FTs (30), or by exofucosylation of the cell surface using purified recombinant α(1,3)-FTs together with the donor nucleotide sugar GDP-fucose (18, 31). In regards to clinical applications, it may be preferable to employ α(1,3)-exofucosylation rather than enforced intracellular α(1,3)-fucosyltransferase gene (“*FUT*”) expression for a variety of reasons, not the least of which is to avoid the potential of alterations in native glycosylation dynamics by introducing a non-physiologic level of the pertinent glycosyltransferase within the Golgi.

The expression of E-selectin ligands controls cellular entry into marrow, skin, and to all inflammatory sites (18). Studies using adoptively transferred regulatory T cells in xenotransplant models of acute graft-vs.-host disease (28, 32) indicate that enforced sLe^X^ expression via α(1,3)-exofucosylation promotes cellular entry into inflammatory lesions (32) and also into marrow (28). Results of both preclinical and clinical studies using human HSPCs (33, 34), and preclinical studies of human mesenchymal stem cells (24) reveal that exofucosylation potently programs cellular delivery to marrow and, notably, preclinical studies show appropriate distribution within marrow (24, 33), and clinical administration of exofucosylated human HSPCs improves engraftment kinetics without any adverse effects (34). Thus, enforcing E-selectin ligand expression on CD19-specific CAR-T cells would drive marrow delivery of these cells. Given the constitutive E-selectin expression in dermal microvessels, it would be expected that exofucosylated CAR T-cells would migrate to the skin, but immunoreactivity would only be triggered in presence of relevant infiltrating tumor cells. However, more generally, because E-selectin expression is characteristically upregulated in tumor endothelial beds (35–46), higher E-selectin binding would increase the ability of CAR-T cells targeting a pertinent malignant cell type to enter relevant lesional tissue [i.e., for solid malignancies (e.g., breast, colon, and lung) and lymphoid malignancies (lymphomas and Hodgkin's disease)]. Beyond enhancing treatment efficacy, the more efficient influx of infused cells into sites where needed would limit collateral damage by lessening cytotoxic T cell accumulations in non-lesional tissue, would allow for decreasing the amounts of infused cells, and commensurately, would trim production costs by diminishing the numbers of expanded cells required to achieve the intended clinical effect. Thus, glycoscience-based strategies can literally steer the pathways for CAR T-cells, providing a roadmap for achieving improved patient outcomes using these cells and other types of adoptive cell immunotherapeutics.

## Author Contributions

The author confirms being the sole contributor of this work and has approved it for publication.

### Conflict of Interest Statement

According to National Institutes of Health policies and procedures, the Brigham & Women's Hospital has assigned intellectual property rights regarding cell surface glycan engineering to RS, and RS has licensed portions of this technology to an entity he has founded (Warrior Therapeutics, LLC), to BioTechne, Inc., and to Mesoblast LTD. RS's ownership interests were reviewed and are managed by the Brigham & Women's Hospital and Partners HealthCare in accordance with their conflict of interest policy.
